# Rapid predictive dosimetry for radioembolization

**DOI:** 10.22038/AOJNMB.2023.74023.1514

**Published:** 2024

**Authors:** Yung Hsiang Kao

**Affiliations:** Department of Nuclear Medicine, The Royal Melbourne Hospital, Victoria, Australia

**Keywords:** Theranostics, Predictive dosimetry Radioembolization, Selective internal radiation therapy, Yttrium-90 microspheres Radionuclide therapy

## Abstract

Economics of today’s busy clinical practice demand both time and cost-efficient methods of predictive dosimetry for liver radioembolisation. A rapid predictive schema adapted from the Medical Internal Radiation Dose (MIRD) method i.e., Partition Model, has been devised that can be completed within minutes. This rapid schema may guide institutions that do not have access to software capable of comprehensive auto-segmentation of lung, tumour and non-tumorous liver, or where rigorous artery-specific tomographic predictive dosimetry is unfeasible for the routine clinical workflow. This rapid schema is applicable to any beta-emitting radiomicrosphere, although absorbed dose-response thresholds will differ according to device. Sampling errors in lung, tumour and non-tumorous liver will compound and propagate throughout this schema. This rapid schema achieves efficiency in lieu of accuracy. The user must be mindful of potentially large sampling errors and assumes all responsibility. Any suspicion of significant error requires the user to revert back to standard-of-care methods.

## Manuscript

 This is a rapid predictive schema devised for liver radioembolization that can be completed in minutes. This schema may guide institutions that do not have access to software capable of comprehensive auto-segmentation of lung, tumour and non-tumorous liver, or where rigorous artery-specific tomographic predictive dosimetry is unfeasible for the routine clinical workflow (1).

 Minimum requirements are planar lung shunt scintigraphy and Tc-99m macroaggregated albumin (MAA) SPECT/CT of the abdomen, inclusive of at least the lower lungs. Non-target Tc-99m MAA deposited in extra-hepatic organs are assumed correctable by interventional techniques, therefore excluded from dosimetry. Residual Tc-99m MAA activity in the syringe and catheter are also assumed negligible.

 By Medical Internal Radiation Dose (MIRD) formalism (i.e., Partition Model) and accepting the false assumption of uniform activity distribution for simplicity (2):

$D_{\mathrm{lung}}=C∙\left(\frac{A_{0}∙\mathrm{LSF}}{M_{\mathrm{lung}}}\right)$

Re-expressed as:

$A_{0}=\frac{D_{\mathrm{lung}}∙M_{\mathrm{lung}}}{C∙\mathrm{LSF}}$

Equation 1

 Where$A_{0}$ is the total prescribed activity (GBq), $D_{\mathrm{lung}}$is the lung mean absorbed dose (Gy), *C* is the radionuclide absorbed dose coefficient (e.g., approximately 50 Gy per GBq/kg for Y-90 radiomicrospheres), LSF is the lung shunt fraction, and $M_{\mathrm{lung}}$ is the lung mass (kg) (2-3). LSF may be calculated from planar scintigraphy or SPECT/CT, keeping in mind that planar scintigraphy tends to over-estimate the true LSF (3). There are also circumstances where conventional LSF formularism may be incorrect (4).

 If thorax Tc-99m MAA SPECT/CT has been performed, or if a separate CT Chest is available, $M_{\mathrm{lung}}$may be calculated from the product of its scan-specific lung mean mass density (g/cm^3^) and its corresponding total lung volume (cm^3^)(3):

$\mathrm{Lung mass density}=\frac{\mathrm{Mean radiodensity}+1000}{1000}$

 Equation 2

 Where mean radiodensity (Hounsfield Unit; HU) has a negative value for lung tissue (3). 

 Alternatively, lung mass may be estimated by linearly scaling the patient’s height to a Standard Man compatible with the patient’s age, ethnicity and gender. The biologic rationale for scaling lung mass according to height, not body weight, has been explained elsewhere in the context of predictive radioiodine prescription (5).

 Tumour-to-Normal Liver Ratio (TNR) formularism is unchanged (2):

$\mathrm{Sampled TNR}=\frac{\mathrm{Sampled tumour count density}}{\mathrm{Sampled non}-\mathrm{tumorous liver count density}}$

 Equation 3

 Which is dosimetrically analogous to:

$\mathrm{Sampled TNR}=\frac{D_{\mathrm{tumour}}}{D_{\mathrm{non}-\mathrm{tumorous liver}}}$

 Where $D_{\mathrm{tumour}}$ and $D_{\mathrm{non}-\mathrm{tumorous liver}}$ refer to mean absorbed doses (Gy) of tumour and non-tumorous liver, respectively. Sampling is a significant source of error, because visual assessment tends to bias towards regions of better count density. Errors are further compounded by MAA as an imperfect surrogate for radiomicrospheres and the natural heterogeneity of its biodistribution. Liver has similar mass density to tumour, therefore differences in mass density are ignored in Equation 3.

 Finally, we introduce a new parameter, the Tumour-to-Lung Ratio (TLR):

$\mathrm{Sampled TLR}=\frac{\mathrm{Sampled tumour counts per gram}}{\mathrm{Sampled lung counts per gram}}$

 Equation 4

 Which is dosimetrically analogous to:

$\mathrm{Sampled TLR}=\frac{D_{\mathrm{tumour}}}{D_{\mathrm{lung}}}$

 Where lung counts per gram (counts/g) is calculated from a sample of lung count density (counts/cm^3^) against its corresponding scan-specific lung mass density (g/cm^3^) using Equation 2. If only abdominal Tc-99m MAA SPECT/CT is available, lung sampling may be performed on the imaged left lower lung, whilst being mindful of potentially significant sampling error. Tumour counts per gram (counts/g) is its count density divided by an assumed mass density of 1.05 g/cm^3^.

 All parameters may be keyed into a spreadsheet as simultaneous equations. To solve for$A_{0}$, the user first decides an initial value of $D_{\mathrm{lung}}$ to be applied into Equation 1 e.g., 20 Gy lung constraint for Y-90 resin microspheres. The user then adjusts $D_{\mathrm{lung}}$ by manual iteration until a balanced prescription for $D_{\mathrm{tumour}}$ and $D_{\mathrm{non}-\mathrm{tumourous liver}}$ is achieved. An example of such a rapid Predictive Calculator is provided in Supplemental Data.

 This rapid schema is applicable to any beta-emitting radiomicrosphere, although absorbed dose response thresholds differ according to device. Sampling errors in lung, tumour and non-tumorous liver will compound and propagate throughout this schema. The user must be mindful of potentially large errors and assumes all responsibility. Any suspicion of significant error requires the user to revert back to standard-of-care methods (1).

## Disclosures

 Y.H. Kao has previously received research funding, speaker fees and is a consultant for Sirtex Medical Limited. No other potential conflicts of interest. This report is unfunded.

## Supplementary Materials



## References

[1] Kao YH, Hock Tan AE, Burgmans MC, Irani FG, Khoo LS, Gong Lo RH (2012). Image-guided personalized predictive dosimetry by artery-specific SPECT/CT partition modeling for safe and effective 90Y radioembolization. J Nucl Med..

[2] Ho S, Lau WY, Leung TW, Chan M, Ngar YK, Johnson PJ (1996). Partition model for estimating radiation doses from yttrium-90 microspheres in treating hepatic tumours. Eur J Nucl Med..

[3] Kao YH, Magsombol BM, Toh Y, Tay KH, Chow PKH, Goh AS (2014). Personalized predictive lung dosimetry by technetium-99m macroaggregated albumin SPECT/CT for yttrium-90 radioembolization. EJNMMI Res..

[4] Kao YH (2014). Dosimetric theory for tumor-to-lung shunt fraction calculation in yttrium-90 radioembolization of noncirrhotic livers. Nucl Med Commun..

[5] Kao YH (2023). First Strike personalized predictive radioiodine prescription for inoperable metastatic differentiated thyroid cancer. Asia Ocean J Nucl Med Biol..

